# Medicines management issues in dementia and coping strategies used by people living with dementia and family carers: A systematic review

**DOI:** 10.1002/gps.4985

**Published:** 2018-09-30

**Authors:** Rosemary H. Lim, Taniya Sharmeen

**Affiliations:** ^1^ Reading School of Pharmacy University of Reading Reading Berkshire UK

**Keywords:** coping strategies, dementia, family carers, medication management, people with dementia

## Abstract

**Objectives:**

Medicines play a key role in the lives of people with dementia, primarily to manage symptoms. Managing medicines is complex for people with dementia and their family carers and can result in multiple problems leading to harm. We conducted a systematic review to identify and model medication issues experienced and coping strategies used by people with dementia and/or family carers.

**Methods:**

Eleven general databases and four systematic review databases were searched. Studies were quality assessed using an established framework and thematically analysed.

**Results:**

Twenty‐one articles were included in this study, and four domains affecting medication use were identified: cognitive, medication, social and cultural, and knowledge/educational and communication. People with dementia reported medication issues in all four domains, but few coping strategies were developed. Family carers reported issues and coping strategies related to the medication and knowledge/educational and communication domains. Common issues with regards to knowledge and communication about medicines remain unresolved. The “voices” of people with dementia appeared largely missing from the literature so were in‐depth understanding of how, whether, and in which circumstances coping strategies work in managing medicines.

**Conclusions:**

Medicines management is a complex set of activities and although current coping strategies exists, these were primarily used by family carers or the person with dementia‐carer dyad. Health and social care practitioners and researchers should seek to understand in‐depth the “mechanisms of action” of existing coping strategies and actively involve people with dementia as co‐producers of knowledge to underpin any further work on medicines management.

**Key points**

1. Our study identified and modelled four key domains that influence the use of medicines: cognitive, medication, social and cultural, and knowledge/education and communication.
2. Current coping strategies exists to manage medication issues but mainly used by family carers or the person with dementia‐family carer dyad.
3. The “voices” of people with dementia appeared to be largely missing from reviewed studies.
4. Health and social care practitioners and researchers should seek to understand in‐depth, the “mechanisms of action” of coping strategies and actively involve people with dementia as co‐producers of knowledge to underpin any further work on medicines management.

## 1 INTRODUCTION

Dementia is a chronic progressive impairment of cognitive function (including memory, reasoning, decision‐making)^1^ caused by various conditions affecting the brain, for example Alzheimer's disease and repeated head trauma.^2^ Globally, dementia presents a critical challenge to both health and social care services.^3^ In the United Kingdom (UK), an estimated 850 000 people currently live with dementia, and, with no cure presently available or on the immediate horizon,^4^ this figure is projected to increase to two million by 2050.^5^ The current cost of dementia in the UK is an estimated £26bn and projected to more than double to £55bn in 2040.^5^ The real impact of dementia, however, extends beyond economics and includes the health, social, and emotional lives of individuals, their families, and wider society.^5^, ^6^, ^7^, ^8^, ^9^

Although there is currently no cure for dementia, medicines play a key role in the lives of people with dementia, primarily to manage symptoms.^10^ Some people with dementia may also be prescribed medication for comorbid health conditions such as Type 2 Diabetes Mellitus or high blood pressure.^11^ Unsurprisingly, given their cognitive problems, managing medication is complex for people with dementia which can result in “medication errors, medication related hospital admissions, and dependence on others to assist with medication management tasks”.^12^ Although non‐adherence to medication is a widespread problem across conditions, ages, and other demographics, and can be intentional or unintentional,^13^ cognitive impairment and dementia have been shown to specifically impact on medication adherence.

With more than two thirds of people with dementia living in the community in the UK, and supported in their daily living by around 670 000 family carers,^5^ there is a need to understand how medicines are being managed or not, in this setting. Such knowledge is important so that health and social care practitioners, and researchers, can focus their work on supporting existing practices and/or designing interventions to enable safe medicines management.

The aim of this systematic review was to identify and model medication issues experienced and coping strategies used by people with dementia and/or family carers.

## 2 METHODS

This systematic review is reported according to the Enhancing Transparency in Reporting the Synthesis of Qualitative Research: the ENTREQ Statement.^14^

### 2.1 Inclusion criteria

Articles were eligible if they (1) reviewed or were designed to investigate specific challenges or barriers to medication adherence among people with cognitive impairment or with dementia or Alzheimer's disease living in their own home; (2) reviewed the role of informal carers or family carers in medication management for people with dementia; and (3) reviewed or designed to explore people with dementia, carers, and/or health care provider's views/perspectives/experiences/relationships or feelings towards medication use, adherence and management, associated barriers and suggested solutions. There were no restrictions on the type of methodology (qualitative, quantitative, or mix of both) reported in the study. Only articles published in peer‐reviewed journals were included. Articles that included study participants under 65 years old were excluded from the review.

### 2.2 Search strategy

Eleven general databases (PubMed, EMBASE, Scopus, PsycINFO, CINAHL, Science Direct, IBSS, Web of Science core collection, The Cochrane Library, Ovid databases, and Google Scholar) and four systematic review databases (The Campbell Collaboration Library of Systematic Reviews, Cochrane Reviews, JBI Database of Systematic Reviews and Implementation Reports, and PROSPERO database) covering a 32‐year time period from January 1986 to June 2018 were searched. T.S. and R.L. searched the databases. A specialised librarian confirmed the comprehensive combination of search terms used. Searches were limited to the English language.

A comprehensive combination of search terms was used in Pubmed and Scopus and adapted for all other databases: (“dementia” OR “Alzheimer's” OR “mild cognitive impairment”) AND (“self‐management” or “patient managed”) AND (“caregiver role*” OR “carer role*”) AND (“medication*” OR “prescription*” OR “pharmaceutical*” OR “drug*” OR “formulation*” OR “dosage form*”) AND (“view*” OR “perception*” OR “attitude*”) AND (“barrier*” OR “challenge*”) AND (“medication administration” OR “medication management” OR “medication use”).

### 2.3 Screening of studies

Two independent researchers (T.S. and R.L.) screened studies for inclusion in the study applying a priori inclusion and exclusion criteria. They screened titles of studies for relevance then abstracts of retained studies. Full texts of retained references were then obtained and screened for eligibility. Reference lists of included studies were searched manually for potentially relevant studies that met the inclusion criteria. Any disagreements were resolved by further discussions and then consensus.

### 2.4 Quality assessment

All qualitative studies were quality assessed using an established appraisal framework^15^ by T.S. R.L. independently quality assessed a proportion of these studies. Any disagreements were resolved by discussion and then consensus. Before conducting a quality assessment of the studies, two screening questions were asked which were directly related to the research aim:

1. Does the paper report on findings from study/studies on medicine adherence or medicine compliance and cognitive impairment (CI)/dementia/Alzheimer's disease (AD) AND/OR Patient/carers experience with medicine adherence or CI or AD or dementia?
2. Is the research relevant to the synthesis topic?

If the answer to either one of these questions was “no,” the study was excluded. Any disagreements were resolved by discussion and then consensus. If both answers were “yes,” the appraisal process then proceeded to the next stage. This next stage involved appraisal of each paper on the following categories: aim, methodology, theoretical perspective, sampling, data collection, data analysis, research partnership relations, justification of data interpretation, transferability, relevance and usefulness, and overall assessment of study.^15^ The appraisal process involved answering yes/no and elaborations on the items contained in the categories assessed. See Table 1 for details of items assessed within each category. Any studies that did not meet key appraisal criteria were excluded from the study (see Table 2).

**Table 1:** Categories and items in the appraisal framework (adapted from Campbell et al, 2011^15^)

Aim	Is there a clear statement of the aims of the research?^a^
Methodology:	Is the methodology (qualitative/quantitative) appropriate for authors stated aims?^a^ In case of a review, is a search strategy identified? Is there a list of keywords provided? Is there a list of inclusion and exclusion criteria provided?
Theoretical perspective	Is a theoretical perspective identified? How would you categorise the theoretical perspective?
Sampling	Is it clear which setting (s) the sample was selected from? (eg, hospital/community) Is it clear why this setting was chosen? Is clear and adequate information given on who was selected? Is it clear why these samples were selected? Is it clear how the sample was recruited? Is the sample size justified by the authors? Is it clear how many people accepted or refused to take part in the research? Is it clear why some participants chose not to take part? Overall, do you consider the sampling strategy appropriate to address the aims?^a^
Data collection	Is it clear where the setting of the data collection was? Is it clear why that setting was chosen? Is it clear how the purpose of the research was explained and presented to the participants? Is it clear how the data were collected and why? (eg, interviews/focus groups, etc.) Is it clear how the data were recorded? (eg, audio/video/notes, etc.) Is there evidence of flexibility or an iterative process in the way the research was conducted? Is it clear who collected the data? Overall, do you consider that the data were collected in a way that addresses the research aims?^a^
Data analysis	Is it clear how the analysis was done? Is it clear how the categories/themes were derived from the data? Is there adequate description of the analysis? Have attempts been made to feed results back to respondents? Have different sources of data about the same issue been compared where appropriate (triangulation)? Was the analysis repeated by more than one researcher to ensure reliability? Overall, do you consider that the data analysis was sufficiently rigorous to address the aims?^a^
Research partnership relations	Is it clear whether the researchers critically examined their own role, potential bias and influence? Has the relationship between researchers and participants been adequately considered?
Findings	Please outline the findings here in as much detail as possible What are the key concepts and interpretations? Please outline in as much detail as possible Were the findings explicit and easy to understand?
Justification of data interpretation	Are sufficient data presented to support the descriptive findings? Are quotes numbered/identified? Do the researchers explain how the data presented in the paper were selected from the original sample? Do the researchers indicate how they developed their conceptual interpretations of what the data contain? Are negative, unusual, or contradictory cases presented? Is there adequate discussion of the evidence both for and against the researchers' interpretations? Overall, are you confident that all the data were taken into account?^a^
Transferability	Is there descriptive, conceptual, or theoretical congruence between this and other work? Are the findings of this study transferable to a wider population?
Relevance and usefulness	Does the study add to knowledge or theory in the field? How important are these findings to practice?
Overall assessment of study	What is your overall view of this study? Would you include this study in the synthesis?^a^

a A “no” answer to these questions were excluded from the study.

**Table 2:** Quality assessment of full‐text articles

Study	Aim	Methodology	Theoretical Perspective	Sampling	Data Collection	Data Analysis	Research Partnership Relations	Findings	Justification of Data Interpretation	Transferability	Relevance and Usefulness	Overall Assessment of Study
1. Ryan A (1998) Medication compliance and older people: a review of the literature^16^												
	√	x	√	x	x	x	x	√	√	x	√	x
2. Fox K, Hinton L and Levkoff S (1999) Take up the caregiver's burden: stories of care for urban African American elders with dementia^17^												
	√	√	√	√	√	x	x	√	√	x	√	x
3. Travis S, Bethea L and Winn P. (2000) Medication administration hassles reported by family caregivers of dependent elderly persons^18^												
	√	√	√	√	√	√	√	√	√	√	√	√
4. Smith F, Francis SA, Gray N et al. (2003) A multi‐centre survey among informal carers who manage medication for older care recipients: problems experienced and development of services^19^												
	√	√	√	√	√	√	√	√	√	√	√	√
5. Cotrell V, Wild K and Bader T. (2006) Medication management and adherence among cognitively impaired older adults^20^												
	√	√	√	√	√	√	√	√	√	√	√	√
6. Francis S‐A, Smith F, Gray N et al. (2006) Partnerships between older people and their carers in the management of medication^21^												
	√	√	√	x	x	x	x	√	√	√	√	x
7. Lindstorm H, Smyth K, Sami S et al. (2006) Medication use to treat memory loss in dementia: Perspectives of persons with dementia and their caregivers^22^												
	√	√	√	√	√	√	√	√	√	√	√	√
8. Arlt S, Lindner R, Rösler A et al. (2008) Adherence to medication in patients with dementia: predictors and strategies for improvement^23^												
	√	√	√	√	√	√	√	√	√	√	√	√
9. Duane F, While C, Beanland C et al. (2011) Making medicines manageable: a culturally and linguistically diverse perspective^24^												
	√	√	√	x	x	x	x	√	√	√	√	x
10. Kaasalainen S, Dolovich L, Holbrook A, et al. (2011) The process of medication management for older adults with dementia^25^												
	√	√	√	√	√	√	√	√	√	√	√	√
11. Campbell NL, Boustani MA, Skopelja EN et al. (2012) medication adherence in older adults with cognitive impairment^26^												
	√	√	√	√	√	√	√	√	√	√	√	√
12. While C, Duane F, Beanland C et al. (2012) Medication management: The perspectives of people with dementia and family carers^27^												
	√	√	√	√	√	√	√	√	√	√	√	√
14. Erlen J, Lingler J, Sereika S et al. (2013) Characterizing caregiver mediated medication management in patients with memory loss^28^												
	√	√	√	√	√	√	√	√	√	√	√	√
14. Wheeler K, Roberts M and Neiheisel M (2013) Medication adherence part two: Predictors of nonadherence and adherence^29^												
	√	√	√	√	x	N/A	x	√	√	x	√	x
15. Prorok J, Horgan S and Seitz D. (2013) Health care experiences of people with dementia and their caregivers: a meta‐ethnographic analysis of qualitative studies^30^												
	√	√	√	√	√	√	√	√	√	√	√	√
16. Gillespie R, Mullan J and Harrison L. (2014) Managing medications: the role of informal caregivers of older adults and people living with dementia^31^												
	√	√	√	√	√	√	√	√	√	√	√	√
17. Poland F, Mapes S, Pinnock H et al. (2014) Perspectives of carers on medication management in dementia^32^												
	√	√	√	√	√	√	√	√	√	N/A	√	√
18. Elliott RA, Goeman D, Beanland C et al. (2015) Ability of older people with dementia or cognitive impairment to manage medicine regimens^33^												
	√	√	√	√	√	√	√	√	√	√	√	√
19. Gillespie R, Harrison L and Mullan J. (2015) Medication management concerns of ethnic minority family caregivers of people living with dementia^34^												
	√	√	√	√	√	√	√	√	√	√	√	√
20. Hudani ZK and Rojas‐Fernandez CH. (2015) A scoping review on medication adherence in older patients with cognitive impairment or dementia^35^												
	√	√	√	√	√	√	√	√	√	√	√	√
21. Smith F, Grijseels MS, Ryan P et al. (2015) Assisting people with dementia with their medicines: experiences of family carers^36^												
	√	√	√	√	√	√	√	√	√	√	√	√
22. Snyder C, Fauth E, Wanzek J et al. (2015) Dementia caregivers' coping strategies and their relationship to health and well‐being: The Cache County study^37^												
	√	√	√	x	√	√	x	√	x	x	√	x
23. Alsaeed D, Jamieson E, Gul M et al. (2016) Challenges to optimal medicines use in people living with dementia and their caregivers^38^												
	√	√	√	√	√	√	√	√	√	√	√	√
24. Campbell NL, Zhan J, Tu W, et al. (2016) Self‐reported medication adherence barriers among ambulatory older adults with mild cognitive impairment^39^												
	√	√	√	√	√	√	√	√	√	√	√	√
25. Aston L, Hilton A, Moutela T, Shaw R, Maidment ID. (2017) Exploring the evidence base for how people with dementia and their informal carers manage their medication in the community^40^												
	√	√	√	√	√	√	√	√	√	√	√	√
26. George N & Steffen (2017) Predicting perceived medication‐related hassles in dementia family caregivers^41^												
	√	√	√	√	x	√	x	√	√	x	√	x
27. Lindauer A, Sexson K, Harvath T A. (2017) Medication management for people with dementia^42^												
	√	x	x	x	x	x	x	√	X	x	x	x
28. Maidment ID, Aston L, Moutela T, Fox CG, Hilton A. (2017) A qualitative study exploring medication management in people with dementia living in the community and the potential role of the community pharmacist^43^												
	√	√	√	√	√	√	√	√	√	√	√	√
29. Smith D, Lovell J, Weller C, et al. (2017) A systematic review of medication non‐adherence in persons with dementia or cognitive impairment^12^												
	√	√	√	√	√	√	√	√	√	√	√	√

Note: √ denotes “yes” and x denotes “no” to the key items appraised (marked ^a^ in Table 1).

### 2.5 Data extraction

T.S. independently extracted the following information from qualitative studies and systematic reviews: authors, year of publication, country where study was conducted, aim of study, study design and methodology, study setting, sample size and participant information/details of studies included (where relevant), and key findings. Abstracting key findings from qualitative studies has been reported to be problematic,^44^ and an approach to resolve this problem is to include all text included under “results” or “findings,” the approach taken in this study. The same approach was taken for systematic reviews. In addition, quotes and results cited in systematic reviews were checked for their source and matched to the list of included studies. Full text articles of references which were not in the list were then obtained, screened for eligibility, quality assessed, and relevant data extracted.

### 2.6 Data synthesis

Due to the heterogeneity of study designs, it was not possible to use a meta‐analysis approach to analyse quantitative findings. A thematic synthesis^44^ with a framework approach^45^, ^46^, ^47^ was used instead. A framework approach, a form of thematic analysis, was appropriate because the research question was focused on a targeted area as opposed to very broad or abstract topics^47^—that of challenges to medication management for people living with dementia and their informal carers. It was also important to ensure the context of each individual study was not lost and a framework approach enables comparison across and within individual cases or studies within the analysis process.^45^, ^47^ In addition, the framework matrix that was formed as part of the analysis process provides a systematic model for mapping and analysing data which is important for the two researchers (R.L. and T.S.) undertaking the review, who are from two different disciplinary backgrounds.^45^ The following steps were undertaken in the synthesis^45^, ^46^, ^47^:

1. Familiarisation: researchers read through extracted data from an initial sample of studies (approximately 3‐5). Key ideas were identified from each of these studies. During this process, recurrent themes were identified.
2. Develop a thematic framework: a framework was then constructed based on the research aims and the recurrent themes identified during the familiarisation process.
3. Indexing: the thematic framework was then applied consistently to all study data.
4. Charting: data from the original studies were then lifted and placed within the appropriate thematic categories within the thematic framework.
5. Mapping: the final stage in the process is to interpret and map the range, polarities, and similarities within the data.

Using a framework approach, the focus was on understanding the breadth and depth of challenges reported in studies rather than the frequency of these problems. The approach to the synthesis did not emphasise weighing data for example through counting how many times a particular challenge was reported. Each reported challenge was considered to be unique and of equal significance. If the same challenge was reported multiple times in multiple papers including systematic reviews, it was still included once in the synthesis.

T.S. and R.L. conducted the analysis through extensive and iterative discussions. Any disagreements were resolved by further discussions and then consensus.

### 2.7 Ethical considerations

Ethical approvals were not required as the study was a systematic review of peer‐reviewed studies.

## 3 RESULTS

### 3.1 Study characteristics

**Figure 1:**
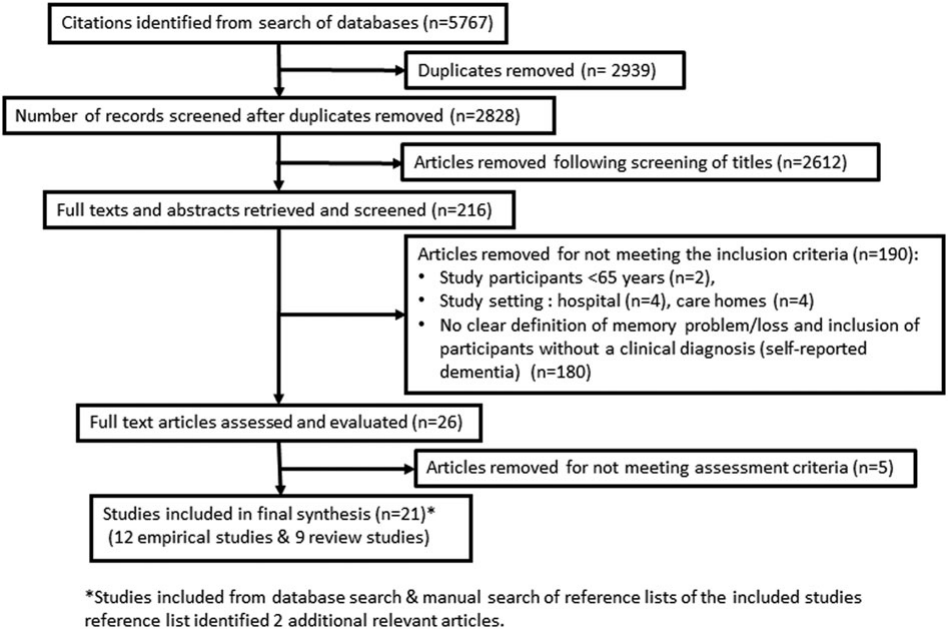
Article selection for inclusion in the study

Figure 1 shows the article selection process. Following quality assessment of studies (shown in Table 2), a total of 21 articles were included in this study: 12 empirical studies and nine reviews (see Table 3). Of the 12 empirical studies, eight used qualitative methods of enquiry,^18^, ^22^, ^25^, ^27^, ^32^, ^34^, ^36^, ^43^ two used mixed methods (a combination of qualitative and quantitative approaches),^19^, ^20^ and two followed cross‐sectional study designs using quantitative methodology.^27^, ^39^ Six studies were carried out in North America (five in the United States and one in Canada), three in Australia, and five studies were conducted in UK. Six of the systematic reviews included both quantitative and qualitative studies,^23^, ^31^, ^33^, ^35^, ^38^, ^40^ two only selected quantitative studies for review,^12^, ^26^ and one was conducted only on qualitative studies.^30^

**Table 3:** Characteristics of studies included in the review

Author (Year), Study Location [Reference]	Study Aim/s	Sample	Data Collection Methods	Data Analysis	Main Findings
Travis et al. (2000), USA^18^	To understand how informal caregivers of older adults manage medication for dependent older adults.	23 informal caregivers of older adults attending adult day care centres. Participants included 22 (96%) female Caucasians.	Semi structured individual interviews, caregiver questionnaire, researcher‐designed medication administration checklist and field notes.	Content analysis.	Caregivers described 122 medication management “Hassles” which were classified into three domains: scheduling logistics, administration procedures, and safety issues.
Smith et al. (2003), UK^19^	To report the number and type of problems experienced by informal caregivers when managing medications for older care recipients.	184 informal carers and 93 associated older care recipients were interviewed at their homes. Participants included 151 (83%) Caucasians, 113 (61%) of which were female.	Cross‐sectional structured individual interviews comprising both closed‐ and open‐ended questions, as well as a validated Caregiver Strain Index.	Closed‐ended questions were analysed using chi squared and Spearman's rank correlation coefficient analysis. Open‐ended questions were thematically analysed.	67% of informal caregivers reported problems with one or more medication management activities including problems with medication supply, administration, making clinical judgements, and communicating with care recipients/ health care professionals.
Cotrell et al. (2006), USA^20^	To examine the relationship between care recipient cognitive status and medication management skills, informal caregiver ability to predict care recipient medication management deficits and corresponding caregiver medication management activities.	47 dyads of informal caregivers and their care recipient, including 27 care recipients diagnosed with Alzheimer's disease and 20 care recipients without Alzheimer's disease.	Survey of care recipients using validated measures: The Medication Complexity Index, the Drug Regimen Unassisted Grading Scale, and the Dementia Deficits Scale, as well as a baseline and follow‐up pill count. Semi‐structured interviews with informal caregivers.	Quantitative data were analysed using descriptive statistics and independent and dependent t‐tests. Interviews were analysed using content analysis.	The majority of informal caregivers of care recipients with Alzheimer's disease (85%) provided assistance with managing medications, as compared with only 30% of those caring for people without Alzheimer's disease. Informal caregivers accurately predict care recipient's medication management abilities and adherence rates (99%). Assistance level is associated with cognitive impairment rather than regime complexity. Success with medication adherence interventions varied.
Lindstorm et al. (2006), USA^22^	To examine the views of Caucasian and African‐American persons with dementia and their caregivers surrounding use of and hopes for existing medications to treat the symptoms of memory loss, willingness to stop medication use at some point in the future, and willingness to try medications that may become available.	19 Caucasian and African‐American persons with dementia and their caregivers (*n* = 19).	Focus groups.	Thematic analysis	Participants (both carer and PLWD) had high hopes for what medications could do to treat memory loss and were optimistic that scientists would find new treatments to significantly affect the course of memory loss in their lifetimes. Participants were generally willing to try hypothetical new treatments, even those with serious side‐effects and high costs. Caregivers and persons with dementia need better information about the likely impacts of medications in order to make informed treatment decisions. Efficacy, side‐effects, cost, and the need for “hope” embodied in concrete actions (eg, taking a medication) must be weighed.
Kaasalainen et al. (2011), Canada^25^	To explore the personal experiences related to medication management of people living with dementia (PLWD), their informal caregivers and assisting health care professionals.	57 English‐speaking participants that included 20 informal caregivers recruited through health care services and Alzheimer's Society, 11 PLWD, 10 nurses, 10 pharmacists and six physicians.	Semi‐structured individual interviews using a grounded theory approach.	Grounded theory.	Management of medicines varied with the severity of dementia. Early stage dementia: medication management characterised by patients' desire to maintain independence, denial of issues or disease, and a refusal to take medications owing to feeling angry. Late‐stage dementia: older adults often refuse medications due to delusional or suspicious thinking, resulting in caregivers assuming responsibility for managing their medications. Reasons informal caregivers assume responsibility for medication management included deterioration in estimative operations (eg, knowledge related to when medication should be taken) and transitional operations (eg, decision‐making ability such as whether to take a medication at a particular time).
While et al. (2012), Australia^27^	To explore the perspectives of PLWD and their informal caregiver living in the community regarding their medication management experiences and to compare their perspectives with people without dementia.	17 participants; eight PLWD were contacted via the Royal District Nursing Service, and nine informal caregivers were contacted via Alzheimer's support groups.	Semi‐structured individual interviews using a grounded theory approach.	Grounded theory.	PLWD are able to sustain self‐ management of their medicines using established routines and strategies. As cognitive changes affect short‐term memory, external strategies and task allocation were taken on by family members to support continuing independence of PLWD. The family member assumed the carer role as their concern for medication safety increased, but this role created stress and was a burden that was unacknowledged by the health professional.
Erlen et al. (2013), USA^28^	To describe informal caregiver medication management in community‐living care recipients with cognitive decline.	91 dyads of informal caregivers and their PLWD care recipient were contacted via geriatric practices, memory clinics, targeted mailing lists, and flyers. Participants included were 85% Caucasian, 10% African American, 1% Asian, and 4% mixed races. The majority of the participants (70%) were female.	Quantitative data included secondary analysis of baseline data from randomised controlled trials of informal caregiver responses using 10 validated measures and an investigator developed medication reconciliation form, as well as the cognitive assessment of the PLWD care recipient.	Quantitative data were analysed using descriptive statistics, two‐sample t‐tests, Mann‐Whitney U‐tests, regression analysis, Spearman's rank order analysis, and Pearson's product moment correlations.	Informal caregivers found medication management challenging and burdensome. The caregiver's age, cognitive ability, depressive symptoms, and perception of their care recipient's behavioural problems can impact on their medication management. Assessing medication management deficiencies requires measuring a number of factors (ie, prescription, acquisition, organization, preparation, and administration and goes beyond just asking whether or not the patient is taking their pills).
Poland et al. (2014) UK^32^	To gain caregivers perspectives on medication issues and how these issues are managed.	9 family caregivers of people with dementia who were current members of the Alzheimer's Society Research Network.	Adapted focus group methodology; the group was facilitated by a specialist mental health pharmacist, using a topic guide developed systematically with carers, health professionals, and researchers.	Thematic and narrative analysis.	Significant themes reported by the carers were related to: (1) medication usage and administration practicalities, (2) communication barriers and facilitators, (3) bearing and sharing responsibility and (4) weighing up medication risks and benefits.
Gillespie et al. (2015) Australia^34^	To examine the medication management experiences of ethnic minority family caregivers of PLWD.	29 family carers.	Three focus groups (22 family caregivers) and seven semi‐structured individual interviews were conducted.	Thematic analysis.	Medication management was a source of stress resulting from the progressive loss of ability of care recipients to manage their own medications; the complexity of the medication regime and the caregiver's lack of trust of the care recipient to safely and effectively manage medications. Strategies to manage medications and avoid conflict with care recipients including being watchful and involving other family members in medication management tasks. Family caregivers indicated that a lack of information and access to support to inform their medication management role added to their stress, which was exacerbated in some cases by limited English proficiency. Supportive factors noted by caregivers included a well‐established relationship with a community pharmacist, involvement of a geriatrician, family support and caregiver support group participation.
Smith et al. (2015) UK^36^	To identify the types of medicines‐related assistance provided by family caregivers of PLWD and the problems surrounding it	A total of 19 semi structured interviews were conducted with 14 family carers and 5 care recipient (PLWD) who were identified through a memory clinic in London.	Semi‐structured interviews.	Framework analysis.	Carers reported challenges included maintaining regular medicine supplies, ensuring adherence to regimens and accessing health professionals. Carers' difficulty in obtaining information and advice about medicines was compounded by their desire to allow the care‐recipient to retain autonomy over their medicines for as long as possible.
Campbell et al. (2016) USA^39^	To compare the frequencies of barriers to medication adherence reported by ambulatory older adults with a diagnosis of mild cognitive impairment (MCI) and ambulatory older adults with normal cognition.	Ambulatory older adults (≥ 65 yrs) with a diagnosis of MCI (96 participants) or normal cognition (104 participants). Interviews were conducted using 17‐item survey includes questions about various domains of barriers including knowledge, financial, behavioural, and physical barriers encountered Over the past 2 weeks.	Cross‐sectional study. Self‐reported beliefs and barriers to medication nonadherence were assessed by items from the Morisky Medication Adherence Survey, the Adherence Estimator, and barriers derived from a systematic review of studies in older adults with cognitive impairment.	Statistical analyses methods including chi‐square test and t tests.	83% reported the presence of at least one barrier to medication adherence and 62.5% reported two or more barriers to medication adherence. The most commonly reported barriers were difficulty remembering the amount or time of each medication to take (49%), difficulty opening or reading prescription bottles (42%), feeling worse when taking medications (29%), and trouble affording medications (26%).
Maidment et al. (2017), UK^43^	To describe and understand the key challenges in medicine management as experienced by people with dementia and their informal carers dwelling in the community and the potential role of community pharmacists in assisting medicine management.	Total 31 participants were interviewed. Among them were 11 informal carers, 4 people with dementia, and 16 HSCPs (four GPs, five nurses, three social care professionals [paid formal carers], and four community pharmacists).	An exploratory qualitative study design that followed consolidated criteria for reporting qualitative studies (COREQ) guidelines	A qualitative framework analysis was undertaken in order to explore the experiences and perspectives of the participants.	Three main themes were identified. Key challenges experienced by informal carers and people with dementia (the caring role, the challenges of the condition), improving medication management in people with dementia (empowerment and communication from health professionals) and the role of pharmacists. The caring role included responsibility for medication management which created both practical problems and an emotional burden. This burden was worsened by any difficulty in obtaining support and PLWDs complex medicine regimen. Study also found that (participants perspective) the process of medicine management could be improved by coordinated and on‐going support from HSCPs, which should focus on the informal carer. Also, medication reviews, particularly when conducted in the home environment, could be helpful.

Author (Year)	Study aim/s	Sample	Data Collection Methods and Analysis	Main Findings
Arlt et al. (2008)^23^	To identify factors associated with adherence to medication in patients with cognitive impairment or dementia, and to discuss strategies for improvement of non‐adherence.	Literature published in PubMed (1987‐2007)	Review of quantitative and qualitative studies	Strategies for facilitating medication adherence in patients with dementia include prescribing as few medicines as possible, tailoring dose regimens to personal habits, and coordinating all drug dosing schedules as much as possible. Technological solutions (automated computer‐based reminding aids, online medication monitoring and telemonitoring) may be helpful for people with mild dementia but it is important to consider actual use. When to introduce assistance with medication management should take into account the person's independence and safety aspects especially with medicines with a narrow therapeutic range. Individual's cognitive status, mood, level of self‐efficacy, and particular living situation should also be considered. No evidence‐based recommendations currently exist.
Campbell et al. (2012)^26^	To conduct a systematic evidence‐based review to identify barriers to medication adherence in cognitively impaired older adults and interventions aimed at improving medication adherence.	13 studies (10 studies met inclusion criteria for barriers to adherence and 3 met inclusion criteria for interventional studies)	Systematic evidence‐based review on quantitative studies	Unique barriers to adherence included understanding new directions, living alone, scheduling medication administration into the daily routine, using potentially inappropriate medications, and uncooperative patients. Two studies evaluated reminder systems and showed no benefit in a small group of participants. One study improved adherence through telephone and televideo reminders at each dosing interval. Successful interventions suggest that frequent human communication as reminder systems are more likely to improve adherence than nonhuman reminders.
Prorok et al. (2013)^30^	To conduct a systematic review of qualitative studies that examined aspects of the health care experience of people with dementia and their caregivers to better understand ways to improve care for PLWD.	46 studies met the inclusion criteria; these involved 1866 people with dementia and their caregivers.	Systematic review of qualitative studies. Meta‐ethnography.	5 major themes were identified: Seeking a diagnosis; accessing supports and services; addressing information needs; disease management; and communication and attitudes of health care providers.
Elliot et al. (2015)^33^	To provide a narrative review of contemporary literature on medicines management by people with dementia or cognitive impairment living in the community, methods for assessing their capacity to safely manage medicines, and strategies for supporting independent medicines management.	A total of 306 articles on medicines management on people with dementia were retrieved and examined for relevance.	Narrative review	As cognitive impairment progresses, the ability to plan, organise, and execute medicine management tasks is impaired, leading to increased risk of unintentional non‐adherence, medication errors, preventable medication‐related hospital admissions and dependence on family carers or community nursing services to assist with medicines management. Self‐report and informant report on ability to safely managing medication may be helpful but can be unreliable or prone to bias. Measures of cognitive function are useful but may lack sensitivity and specificity. Direct observation, using a structured, standardised performance‐based tool, may help to determine whether a person is able to manage their medicines and identify barriers to adherence such as inability to open medicine packaging.
Gillespie et al. (2014)^31^	To explore published literature that describes what is known about the role of informal caregivers as they manage medications for older adults and/or people living with dementia residing in the community.	10 articles reviewed	Narrative review.	The role of informal caregiver is complex and often made more difficult because of increasing medication regimen complexities, aspects of the relationship between the caregiver and the care recipient, health care system practices, and a lack of information and/or training available to the informal caregiver, especially when caring for people living with dementia.
Hudani et al. (2015)^35^	To explore aspects of medication adherence among older patient with cognitive impairment or dementia.	42 articles and 2 conference proceedings were reviewed	Scoping review	The prevalence of non‐adherence ranged from 2% to 59%. The most common assessment tool was self‐reported adherence (32%). Barriers to adherence were cognitive impairment, non‐Caucasian race, poor communication with prescribers, lack of social support, and increased pill burden. Interventions to improve adherence included alternate dosage forms, and multi‐compartment pillboxes.
Alsaeed et al. (2016)^38^	To explore and ascertain the issues which affect optimal medicines use from the viewpoints of people living with dementia and their caregivers (informal unpaid, ie, family members and formal or paid professionals, ie, nurses), in the community and care home settings.	16 studies were reviewed	Literature review	Six broad themes were identified (organisation and scheduling logistics, administration procedures and health literacy, impact on caregiver, impact on PLWD, partnership between caregiver and PLWD and interface with formal care) together with suggested recommendations (promoting communication between patient‐carer dyad and HCPs, provision of clear information, deliver tailored support and training, and better services etc.)
Aston et al. (2017)^40^	To explore and evaluate the existing literature on how people living with dementia manage their medication; to assess understanding of medicines management from an informal caregivers viewpoint; and to understand the role of HCPs in assisting medicine management.	9 articles were reviewed	Mixed studies review using a convergent synthesis approach	Four themes were generated from the synthesis: (1) effects of dementia on medicines management and the use of adherence aids, (2) impact on informal carers, (3) knowledge of medication as an aid to adherence, and (4) health care professionals' understanding of medicines management in people with dementia. These themes were found to affect management of medication, in particular adherence to medication.
Smith et al. (2017)^12^	To explore the relationship between medication non‐adherence and specific cognitive domains in persons with CI, and to discover determinants of medication non‐adherence	15 articles were reviewed	A systematic review	This review found poor cognitive function as a risk factor of medication non‐adherence. It also highlighted the importance of caregivers in assisting with medication adherence or interventions to improve medication adherence. Review also suggested that clinicians should be aware of the negative effect global cognitive impairment has on medication adherence and consider screening patients where impairment is indicated.

The key characteristics and main findings from included studies are presented in Table 3. In eight of the 12 empirical studies, the study design included people with dementia and family carers as participants.^19^, ^20^, ^22^, ^25^, ^27^, ^28^, ^36^, ^43^ However, only four of these studies interviewed matching dyads (people with dementia with their family carers).^19^, ^20^, ^36^, ^43^ Seven studies included people with dementia.^20^, ^22^, ^25^, ^27^, ^36^, ^39^, ^43^ The study design in these studies included semi‐structured interviews (*n* = range 4‐11 participants),^25^, ^27^, ^36^, ^43^ self‐reporting of beliefs and barriers to medication non‐adherence (*n* = 96),^39^ assessment of performance on medication management tasks (*n* = 27),^20^ and focus group (*n* = 19).^22^ One study^39^ compared the frequencies of barriers to medication adherence between people with dementia and adults with no cognitive issues.

### 3.2 Key domains affecting medication use

**Figure 2:**
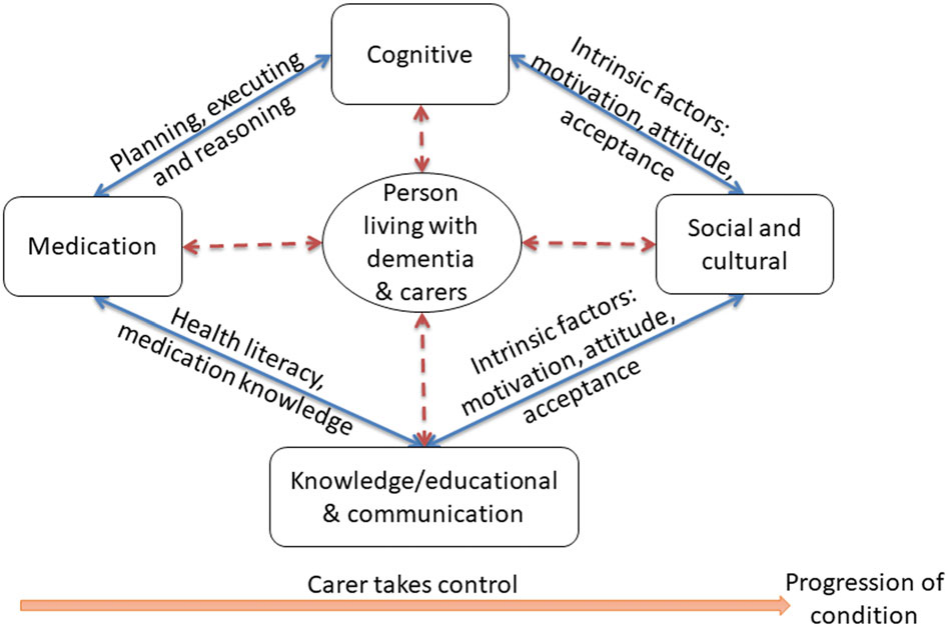
Domains and factors affecting medication use [Colour figure can be viewed at http://wileyonlinelibrary.com]

Secondary analysis based on the synthesis of primary accounts reported in the included studies identified four domains affecting the use of medication as reported by people with dementia and/or their carers: cognitive, medication, social and cultural, and knowledge/educational and communication (see Figure 2 and supporting data and quotes in Table 4).

**Table 4:** Reported challenges and solutions to medication management as described by people living with dementia and family carers

Domain	Person Living with Dementia		Family Carer	
	Reported challenges	Practical solutions	Reported challenges	Practical solutions
Cognitive^23^, ^25^, ^27^, ^32^, ^34^, ^43^	Deterioration of the ability to plan, organise, and execute medicine management tasks/ (deterioration of cognitive and functional ability) Forgetfulness and confusion, lack of insight Forgetful caregiver	Accepting assistance with obtaining, storing, opening, and timely administration of dosage, given up/transfer control of medicine management and responsibility to family carer.	Communicating with care recipient/communicating with confused uncooperative care recipient Suffering from cognitive impairment themselves	Using visual aids and/or external memory reminders such as diaries, alarms, activity planner etc. and hiding medicines in PLWD's food
Exemplar	*“I never really think about my medications … No. I just take them when they're there”*. PLWD^25^ *“I find organising things in the short term, I find it difficult”* PLWD^43^ *“Well um, it's just simply forgetting, finishing your meal and not remembering to take the medications following it”. I, I have um, if somebody is here, they will quite often remind me”*. PLWD^25^ “I forget the name but I know what I mean. I'm forgetting something. I get very Worried …. there'd be occasions that I've *sort of missed out [my medication*” PLWD^27^	*“Sometimes I think there's a kind of relief that somebody's taken it over”* PLWD^27^	*“So my husband, he didn't accept the medications, he wouldn't take the medications”* Carer^34^ *“My husband he remembers, he takes his own medication what he like but he only takes the blood pressure tablets because the other tablets he thinks do not do anything to him so it's no worth to take it.”* Carer^34^ *“[If] she doesn't want it, she won't swallow it, and then sometimes with the puffer she will blow it the other way … you know she'll spit up pills. Sometimes I know she hasn't taken the medication because I find it on the floor. Gotta watch her”*. Carer^35^	*“So, we started hiding the medication in food, we crushed it … put it in some yoghurt. Some of them weren't crushable so we put them whole into liquorice or something that has a strong taste over powering the other one”*. Carer^34^ *“then I have to crush it up, I have to mix it with banana and she seems to swallow that”* Carer^35^
Medication^23^, ^25^, ^27^, ^32^, ^33^, ^36^, ^38^, ^40^, ^43^	Risk of experiencing medication errors (underdoes, overdoses, unintentional non‐compliance etc.) Introduction of new medications Learning new medication systems or regimen Complexity of the medicine regimen/the number and frequency of medicines	Use of medicine aids (pill box, Dosett box) Use of internal and external memory strategies (medicine regimen that is linked with person's daily routine, use of reminder aids and strategies)	Maintain continuous supply of medicine Order prescription(s) Collect prescription(s) Collect medicine(s) from different places Monitor the need of further supply Monitor delays in issuing prescription Transport care recipient to and from the surgery Unsuitable opening times (surgery and pharmacy) Return visits to surgery to query prescription details and accuracy Incomplete supplies of medications from pharmacy Possible errors in dispensing and labelling that require return visit Waiting times in surgery and pharmacy Careful organization and vigilance of medicine Assisting with administration Open containers (when PLWD had problem with the packaging) Remind care recipient when to take medication/giving medicine on time Help with practical administration of medication (split tablets, administer eye drops, apply creams etc.) Schedule multiple medications throughout the day Giving medication to a confused or uncooperative person To administer a painful, embarrassing, or noxious medication Knowing how to make up missed doses New medications or learning new medication systems Number and frequency of medications Overall supervision of medicine use Staying constantly vigilant for problems related to medicine use Caregivers indicated that they were “on duty” for much of the day.	Solutions to maintain continuous supply of medicine Establish routine for managing medications Use of systems and practices that allows Sending or faxing prescription/repeat prescription from surgery to the pharmacy or to the PLWD/care recipient's home. Online repeat prescription requests Home delivery of medications Emergency supply from pharmacist until repeat prescriptions was available. Practical solutions for assisting with medication administration Use of internal and external memory strategies Associate medication‐taking with a routine Remove medication from containers in advance and place them somewhere visible/familiar Use of compliance aids Use of external memory (taking notes, making lists, using alarm clocks/timers) Simplify dosing regimens Optimising medication types (eg,, liquids)
Exemplar	“*Something new added can really throw my whole routine off if when inhalers got added. Um, and for a while I was taking a fancier system of inhalers following sort of a breathing crisis. And then it was quite complicated, you were supposed to take it at different times of the day. All that and then the ones that you put, you swallow and it was just, it was so complex for me …*. *that's very hard too”*. PLWD^25^	*“I take the seven things and I take them all in the morning. it's the first thing I do and then I don't have to worry.”* PLWD^27^ *“got a noticeboard as well as little reminders … .the nice bright yellow top on‐top of your fridge … I leave the flap open and take the pill out for the day and most of the time I can remember what day it is because of what we're doing. If that flap is open I've taken the pill.”* PLWD^27^ *“you see what's made such a difference to me is the Webster‐pak. It's just at the right time for me and I feel safe as houses.”* PLWD^27^	*“One day she might forget, another day she might take two lots, over‐medicating. But when she was over‐medicating you could tell*. .. *because she wasn't with it*. .. *she wasn't as bright or she was sleepy or very incoherent.”* Carer^27^ *“They [pills] seem to get stuck in his mouth, and if he tries to chew them then that doesn't always work”* Carer^27^ *“It is actually coordinating all the bits and then sometimes they [the Dr's] will write you three months or four months or five or six. But there is no consistency to it. So, unless you monitor it and watch it carefully yourself, you are going to end up running out of things*. Carer^27^ *“*I *think the main problem with the medication was having to go and get it and remembering, “Oh, there's only that many left of that particular one, I'd better order some more.” And I seemed to be up and down there twice a week.”* Carer^43^ *“We would get up to the pharmacy. I'd get half of what I needed and then I find half of it is not there. It was such a waste of time and it was also inconvenient. I was back and forth and up to the pharmacy”*. Carer^25^ “The medication was delivered to my mum by the pharmacy. But suddenly they stopped. I live the other side of London. They did not mention this earlier. So, I had to spend another day phoning around to get everything done. I had come not to worry too much if she missed a day *of the ramipril or aspirin because these pills were more preventative. But I didn't want her to miss the Aricept [donepezil].”* Carer^36^	*“, … so it's better if I crush them [pills] and get them in his applesauce and get them down that way … I would say that drugs that were designed to be taken by people in this condition would be wonderful.”* Carer^27^ *“… After that I wrote down in my agenda when to get a new prescription.”* Carer^36^
Social and cultural^23^, ^25^, ^27^, ^33^, ^34^, ^36^, ^43^	Living alone Lack of help, adequate supervision and appropriate support at home Limited caregiver availability	Accept assistance with obtaining, storing, opening and timely administration of medication	Stress caused by carer responsibilities	Respite, time off from constant care responsibilities Use of temporary replacement carer (other family members/close friends)
Exemplar	*“I need to call my son … the GP put me on furthermore tablets. I don't know what they are for or what they are.”* PLWD^34^ *“When she lived alone and had to take her blood pressure tablets on her own and I came in the afternoon, the tablet was still in the dosage box and I didn't know what to do. I did not know if it could hurt her to give the tablet at that moment or if I had to wait until the next morning.”* PLWD's challenge reported by family Carer^36^	“It's good with [my son] because he understands and all and he knows sort of what to do so I'm quite happy with him taking care of my *medication.”* PLWD^27^ “*my son goes down to the* *pharmacist*… *and he gets them.”* PLWD^27^ “*Well my family need to* Know all about it … because there is no point telling me, so they have to know *everything”*PLWD^36^	*“Well, this caused me a lot of burden and stress in the beginning. Like, really max. Even though I actually understood what I was doing.”* Carer^25^ *“I do that [fill the tablet box] every Sunday morning because I get very tired in the afternoon. I have to do it when I'm really functioning. I have so much medication and that's why I think I have to do things when my mind is really clear.”* Carer^25^	*“If you're dealing with a dementia person, get a neighbour or friend to come and stay for a couple of hours. The Alzheimer's Society you can go to meetings for the caregivers, you can bring your loved ones there, they are in a room with just a door separating you and they do things with them … it is crucial that you get support not just pills from the doctor and send you home –you have these people at home with you, you care for them and to have other people that you can talk to going through it, no one else understands, they just don't.”* Carer^25^
Knowledge/education and communication^23^, ^25^, ^27^, ^30^, ^33^, ^34^, ^36^, ^43^	Difficulty to know how to take new medications Acquire knowledge of treatment indication and directions	None reported	Communicate with health care professional (including information gathering) Ability to understand and make clinical judgements and be aware of medicine safety issues. Know how to give medications safely Know when to hold, alter, or discontinue a medication Recognise side/adverse/toxic effects of medicines Know how to recognize and respond to an emergency	Make extra efforts to build a communicative relationship with health care professionals Develop a contingency plan for example, a folder containing medication history and current medicine regimen Preparedness on how to deal with adverse situation or emergency
Exemplar	*“I wouldn't know which ones to take, there's too many of them.”* PLWD^43^		“Don't forget that the clinician and pharmacist can have little or no *understanding of the practicalities.”* Carer^32^ *“I just dread that if I would make a mistake*. .. *I have a lot of tablets so I don't want to make a mistake with my own as well as my husbands.”* Carer^27^	“*After months I got it absolutely perfectly balanced with a lot of help from the District Nurse …*.*she went way beyond what she should have done …*. I *will be eternally grateful to her.”* Carer^32^ “*I think I'm lucky in a way that I've been able to use experiences that I've gained along the way and to put them into practice now. I think a lot of people are not able to do that.”* Carer^27^ “*I've also put all that [medication information] and mine on my mobile* *phone in a notes file.”* Carer^27^

The cognitive domain includes factors such as the impairment of cognition, typically involving problems related to memory and at least one other cognitive domain (language, visuospatial, executive function) as reported by people with dementia and/or carers.^12^, ^23^, ^25^, ^27^, ^34^ In the medication domain, factors relating to obtain and use of medicines,^27^, ^36^, ^38^, ^43^ risks of experiencing medication error,^27^ concerns about medication and patient safety,^27^ and the complexity of the medicine regimen^25^, ^27^ were identified. Factors relating to the lack of appropriate support and help,^25^ caregiver availability,^25^, ^40^ and living alone^23^, ^25^, ^33^ formed part of the social and cultural domain. The knowledge/educational and communication domain represent the factors related to difficulty in understanding complex regimen^27^, ^36^ and ability to make clinical judgement in times of need.^27^, ^36^

People with dementia can be affected by the changes in any of these four domains due to the inter‐relationships among the domains. A decline in cognitive ability affects people with dementia's ability to plan, execute, manage, and organize medicine‐related tasks.^23^, ^33^, ^34^ Issues in the cognitive domain can result in confusion and lack of insight^27^, ^34^, ^36^ which in turn leads to risks of experiencing medication errors (underdose, overdose, wrong drug), unintentional non‐adherence, or adverse events^23^, ^27^, ^34^ (in the medication domain). This demonstrates a link between the cognitive and medication domain. Health literacy and ability to understand and acquire medicine knowledge about treatment indication and directions have a direct impact on medication use^27^, ^36^, ^40^ showing a link between the knowledge/education and communication domain, and the medication domain. The inter‐relationships between socio‐cultural, education/knowledge, and cognitive domain are linked with intrinsic factors such as motivation (to learn new medication regimen/treatment)^27^, ^30^, ^36^; attitudes, beliefs, and desire to maintain independence and self‐management of medicines^27^; motivation to acquire knowledge or awareness/insight into cognitive and functional impairment and acceptance of the disease and willingness to accept help/use of adaptive strategies.

### 3.3 Issues related to medication use and coping strategies

Table 4 provides a summary of specific issues to medication use as reported by people with dementia and family carers alongside any coping strategies reported to address them. Exemplars in the form of quotes from included studies are also included in Table 4 to show how challenges, coping strategies, and the overall domains affecting medication use were derived. People with dementia reported issues in all four domains (cognitive, medication, social and cultural, and knowledge/educational and communication). However, there were few solutions that they have employed to aid medication use. Most of the challenges reported by family carers related to the medication and knowledge/educational and communication domains; these were matched with corresponding solutions. Table 4 also highlight challenges faced by the dyads and the reactive strategies that they have used are similar in nature (ie, cognitive and maintaining continuous supply of medication). There were however challenges faced by both people with dementia and family carers that remain unresolved, eg, managing challenges relating to communicating with people with dementia (for family carers) and knowledge about indication of prescribed medication (for people with dementia).

## 4 DISCUSSION

To our knowledge, this is the first systematic review that identified and modelled a range of system factors influencing the overall management use of medicines and matching coping strategies within four inter‐related system domains, as expressed by people with dementia and/or family carers. Although there are existing systematic reviews investigating medication issues, they have focused specifically on medication non‐adherence,^26^ the overall health care experiences of people with dementia and their caregivers (not specific to medication management),^30^ and the relationship between medication non‐adherence and cognitive domains (eg, memory and executive functioning).^12^ This current study modelled medication management issues and challenges within the wider medication management system (not restricted to the person or dyad, including but not restricted to factors affecting/associated with medication non‐adherence, health care system‐level issues related to medicines management), and mapped the interconnectivity among/between other system domains. It is important to present medication issues from a systems view rather than individual parts/domains because it is now widely acknowledged that interventions developed to manage medicines require a whole systems view.

Our study confirms that medication management comprised a complex range of activities for the person with dementia‐carer dyad. This finding is in line with existing studies that highlighted medication management as a complex extensive concept that requires a combination of management and organization skills, clinical knowledge, and understanding of medicine safety.^31^, ^34^, ^43^

Unsurprisingly, our study identified wide‐ranging system issues relating to medicines management and some corresponding coping strategies, as experienced by people with dementia and/or family carers. These were categorised into cognitive, medication, social and cultural, and knowledge/educational and communication domains. Analysis of the meta‐data from the studies included in this review identified similarities between people with dementia and family carers in their experiences of difficulties surrounding medicines management. Whilst these similarities could be attributed to “ageing” [while], other studies suggested the family carers' role as advocates for people with dementia to be a key factor.^23^, ^38^, ^39^, ^48^

The complexities of managing medicines increases with the progression of dementia. Most of the strategies employed by people with dementia and/or family carers at the initial stages of dementia were reactive in nature. Family carers played a crucial compensating role in managing medicines as the disease progresses, and they are developing proactive strategies to manage medicines.^27^ Progressive deterioration of the person with dementia's sight, cognition, and dexterity poses challenges. Declining cognitive function makes understanding and retaining medication knowledge or remembering and learning new medicine regimen difficult. In these circumstances, it appears that people with dementia transfer their control of managing medication to their family carer.^20^, ^23^ The family carer eventually takes on the role of organization and management of medicines, and the responsibility of administering the prescribed medication which contributes to stress and a reduction in their quality of life.^8^, ^27^, ^49^, ^50^, ^51^ There appears to be no available data on how people with dementia give up their control, how the transition happens, and what types of challenges, eg, emotional (giving control to someone else, adjusting with independence/sacrificing independence) and socio‐cultural factors a person faces and what type of coping mechanism are used. In addition, there is very little documented in the published literature on the challenges family carers faced during this transition phase especially the emotional costs, the communications gaps, and complex ethical decision‐making burdens they had to take on behalf of the people with dementia.

With declining cognitive ability, people with dementia are less likely to use internal memory strategies to aid with medication administration.^23^ Studies included in this review^25^, ^27^, ^32^, ^36^ found that recall of repetitive tasks such as medication administration could be supported by connecting the activity to environmental cues, a strategy that was mentioned by both people with dementia and their family carers. Dose administration aids, specifically the Webster‐pak, are common external memory strategies for older people^52^, ^53^, ^54^, ^55^ and people with dementia used these more frequently than older people.^27^ Whilst there is documented evidence that carers have developed coping strategies to help with the organisation of medication for example in ordering, collecting, and storing of medication, there are still gaps to managing cognitive challenges, eg, communicating with people with dementia or communicating with confused uncooperative care recipient highlighting the need to investigate these in future studies.

There appeared to be no coping strategy for managing medication issues such as knowledge about indication of prescribed medication in this study. It is also interesting to note that none of the studies in this review reported support or coping strategies for people with dementia to self‐manage their medicines. However, other supporting strategies are in place such as de‐prescribing (withdrawing of medications) which has been undertaken with some success^56^, ^57^ and the use of home care agencies to help the person with dementia, mainly with their daily living activities with limited roles in managing medicines. There is also greater recognition and advocacy in involving people with dementia in co‐design^58^, ^59^, ^60^ although such approaches focusing on managing medicines is scarce. This presents a need for further research into this area given that many people with dementia live alone in their own homes.

Despite current knowledge of existing coping strategies, there appears to be little insight or in‐depth understanding as to how, whether, and in which circumstances these strategies work for people with dementia and/or family carers and any adjustments they make to manage medicines. For example, there is limited literature reporting how and when people with dementia and/or family carers decide to switch from internal memory strategies to external adaptive strategies and, whether and in which circumstances these strategies are effective in managing medication. Future studies are needed to investigate in‐depth the “mechanisms of action” of medication management strategies currently employed by people with dementia and/or family carers using realist approaches^61^, ^62^ to inform recommendations for managing medicines safety in people's own homes.

Despite the inclusion of people with dementia in some of the studies reviewed in this study, their perspectives towards medicine use such as medication issues and coping strategies were largely missing from the study results; their “voices” did not seem to be represented compared with those of family carers. This is puzzling and could be due to multiple factors such as the study design and how research was conducted with people with dementia. Several studies report efforts to address the issue of study design by carefully considering inclusive approaches that enables people with dementia, largely those with early stages of dementia, to contribute to research in a meaningful way.^63^, ^64^, ^65^, ^66^ However, long‐standing ethical and methodological issues such as providing informed consent, doubts about the reliability of their accounts, and communication problems^67^, ^68^, ^69^, ^70^ in those with later stages of dementia still require further discussion and testing. Active and real involvement of people at different stages of dementia in research is crucial to enable evidence‐based practice or interventions designed to improve management of medicines by people with dementia and/or carers living in their own homes. Future studies should strongly consider taking forward suggestions for including people with dementia in research, such as those proposed by Alzheimer's Europe^70^ that advocates an inclusive approach to research by entering into a collaborative relationship with people with dementia and their advocates and also providing the necessary infrastructure to enable such collaboration to take place effectively. Research conducted within this culture and practice could be more conducive and effective in exploring people with dementia's perspectives and lived experiences on medication related beliefs, strategies, preferences, and routines, along with the needs of carers, so that an individualised and person‐centred approach can be developed and used in practice.

### 4.1 Study limitations

Literature searches were limited to the English language and excluded grey literature. The studies reviewed were also largely exploratory in nature, and few specified any theoretical underpinnings to their study design. The two researchers involved in the analysis were from pharmacy/health system/human factors and bio‐cultural and medical anthropology backgrounds which may not represent a wide range of perspectives when interpreting findings from studies. To address this limitation, the findings of the systematic review were also discussed with a group of stakeholders (who formed the project advisory group of the wider study within which this systematic review was conducted) that included people living with dementia, family carers, community pharmacist, general practitioner, charity representative, and academics, and presented at an international dementia conference.^71^ Overall, the findings resonated with people's personal experiences and further comments were made regarding the lack of appreciation of the “social” domain in everyday clinical practice. Although individual issues identified in this study were not new, feedback received highlighted the systematic, conceptual, and visual way in which medication management issues/domains were presented and the interconnection between domains to be an important addition to the current body of knowledge.

## 5 CONCLUSION

In this study, we identified and modelled wide‐ranging system factors influencing the use of medicines, as expressed by people with dementia and/or family carers. Medicines management is a complex set of activities and although current coping strategies exists to manage medication issues, these were primarily used by family carers and/or the person with dementia‐carer dyad. There also appear to be few strategies to support cognitive issues such as communication and understanding indications of prescribed medicines. In‐depth understanding of how, whether, and in which circumstances coping strategies work for people with dementia and/or carers were largely missing. Although some studies have included people with dementia in their studies, the real “voices” of people with dementia appeared to be largely missing from the literature. Future studies should (1) investigate in‐depth the “mechanisms of action” of coping strategies employed by people with dementia and/or carers and (2) include people with dementia as co‐producers of research knowledge (ie, doing research collaboratively as opposed to doing research on people with dementia) to underpin further work on developing interventions to enable safe medicines management by people with dementia and/or the person with dementia‐carer dyad in their own homes.

## AUTHORS' CONTRIBUTIONS

R.L. made a substantial contribution to the conception and design of the work, the interpretation of data, drafting of the paper, and supervision of the study. T.S. made a substantial contribution to the design of the work, the acquisition and interpretation of data, and drafting of the paper. All authors approved the final version of the paper.

## FUNDING

The study was part of a study funded by the Wellcome Trust Seed Award in Humanities and Social Science awarded to R.L. (grant number 108320/Z/15/Z). The funders had no role in the design of the study, the collection and analysis of the data, or in the preparation of the manuscript.

## CONFLICT OF INTEREST

None declared
